# Electrical defects of the transverse‐axial tubular system in cardiac diseases

**DOI:** 10.1113/JP273042

**Published:** 2017-01-17

**Authors:** Claudia Crocini, Cecilia Ferrantini, Raffaele Coppini, Leonardo Sacconi

**Affiliations:** ^1^ European Laboratory for Non‐Linear Spectroscopy50019Sesto FiorentinoItaly; ^2^ National Institute of OpticsNational Research Council50125FlorenceItaly; ^3^ Division of Physiology, Department of Experimental and Clinical MedicineUniversity of Florence50134FlorenceItaly; ^4^ Division of Pharmacology, Department ‘NeuroFarBa’University of Florence50139FlorenceItaly

**Keywords:** action potential, calcium release, cardiomyocyte, T‐tubules

## Abstract

Electrical excitability is an essential feature of cardiomyocytes and the homogenous propagation of the action potential is guaranteed by a complex network of membrane invaginations called the transverse‐axial tubular system (TATS). TATS structural remodelling is a hallmark of cardiac diseases and we demonstrated that this can be accompanied by electrical defects at single T‐tubular level. Using a random‐access multi‐photon (RAMP) microscope, we found that pathological T‐tubules can fail to conduct action potentials, which delays local Ca^2+^ release. Although the underlying causes for T‐tubular electrical failure are still unknown, our findings suggest that they are likely to be related to local ultrastructural alterations. Here, we first review the experimental approach that allowed us to observe and dissect the consequences of TATS electrical dysfunction and then propose two different strategies to unveil the reasons for T‐tubular electrical failures. The first strategy consists in a correlative approach, in which the failing T‐tubule identified with the RAMP microscope is then imaged with electron microscopy. The second approach exploits the diffusion of molecules within TATS to gain insights into the local TATS structure, even without a thorough reconstruction of the tubular network. Although challenging, the local electrical failure occurring at single T‐tubules is a fundamental question that needs to be addressed and could provide novel insights in cardiac pathophysiology.

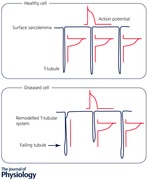

**Abbreviations**
AOD: acousto‐optic deflector
AP: action potential
CICR: calcium induced calcium release
cTnT: cardiac troponin T
DHPR: dihydropyridine receptor
ECC: excitation–contraction coupling
EM: electron microscopy
HCM: hypertrophic cardiomyopathy
HF: heart failure
NCX: Na^+^–Ca^2+^ exchanger
RAMP: random access multi‐photon
RyR2: ryanodine receptor 2
SERCA: sarcoplasmic reticulum Ca^2+^‐ATPase
SR: sarcoplasmic reticulum
SS: surface sarcolemma
TATS: transverse‐axial tubular system
TPF: two‐photon fluorescence
TT: T‐tubule
VSD: voltage‐sensitive dye

## Physiology of the transverse‐axial tubular system

Mammalian ventricular cardiomyocytes are characterized by an extensive system of deep invaginations of the sarcolemma called the transverse‐axial tubular system (TATS) or T‐tubules (Lindner, 1957; Forssmann & Girardier, 1970; Forbes *et al*. 1984; Franzini‐Armstrong *et al*. 1999; Brette & Orchard, 2003; Ferrantini *et al*. 2013). The TATS architecture consists of transverse components profiling Z‐lines, and longitudinal or axial elements running from one Z‐line to the next. The cardiac T‐tubular diameter is about 100–300 nm, significantly larger than T‐tubules in skeletal muscle (20–40 nm). The functional role of T‐tubules is to ensure the rapid propagation of the action potential (AP) to the cell core triggering Ca^2+^ release from the sarcoplasmic reticulum, and eventually the contraction of myofilaments (excitation–contraction coupling, ECC). During the plateau of each AP, Ca^2+^ enters the cell through depolarization‐activated Ca^2+^ channels, known as dihydropyridine receptors (DHPRs). Ca^2+^ binds to the ryanodine receptor 2 (RyR2) inducing a massive release of Ca^2+^ from the sarcoplasmic reticulum (SR) (calcium induced calcium release, CICR). The combination of external Ca^2+^ influx and release from the SR raises the free intracellular Ca^2+^ concentration allowing Ca^2+^ to bind to troponin C and switch on the contractile machinery. The SR Ca^2+^‐ATPase (SERCA) and sarcolemmal Na^+^–Ca^2+^ exchanger (NCX) lower the intracellular Ca^2+^ concentration allowing muscle relaxation (Bers, 2002). Immunochemistry studies have revealed the crucial role of T‐tubules in ECC. In fact, key sarcolemma proteins are localized predominantly in the T‐tubular membrane (Brette & Orchard, 2003; Pasek *et al*. 2008a), with respect to the surface sarcolemma (SS). Dyads are also reported to be more abundant in the TATS membrane than SS, about 75:25 in rat ventricular myocyte (Brette *et al*. 2006). An elegant study exploiting the restricted diffusion space within the T‐tubular lumen probed the current distribution in cardiac sarcolemma. By applying a rapid change of the bathing solution, some currents show an initial fast change, attributable to the channels located in the SS, followed by a slower phase representing the contribution of channels in the TATS (Shepherd & McDonough, 1998). Based on these experiments, about 64 % of Ca^2+^ current (*I*_Ca_) is distributed in the TATS. Alternatively, currents distribution has been studied by selectively disrupting the T‐tubular network from SS. This technique, named detubulation, has been described and extensively validated in isolated ventricular myocytes (Kawai *et al*. 1999; Brette *et al*. 2002). Comparing the loss of cell capacitance (a function of membrane area) with the loss of the density of membrane currents after detubulation, it has been demonstrated that many membrane currents, including *I*_CaL_ and *I*_NCX_ are predominant in the TATS (Yang *et al*. 2002; Brette *et al*. 2004). However, the TATS architecture and thus membrane currents distribution are species and chamber specific. In atria, for instance, the TATS is much less developed (Bootman *et al*. 2006; Dibb *et al*. 2013). A recent work reported that mouse and human atrial TATS is composed of voluminous axial tubules (ATs) that are connected to the surface membrane through sparse transverse components and are coupled to junctional highly‐phosphorylated RyR2 clusters, allowing rapid Ca^2+^ transient onset in the cell‐core (Brandenburg *et al*. 2016).

## Morphological alterations of the diseased TATS and implications on Ca^2+^ release synchrony

Loss and disorganization of the TATS have been found in several pathological conditions, unveiling the paramount role of the tubular network in cardiac physiology. Particularly, studies performed on human ventricular tissue from patients with cardiac hypertrophy or heart failure (HF) have identified pathological alterations of the TATS for the first time (Maron *et al*. 1975; Schaper *et al*. 1991; Kostin *et al*. 1998; Kaprielian *et al*. 2000). Moreover, disease‐related TATS structural abnormalities in ventricular cardiomyocyte have been also observed in several animal models and, generally, include:

1. reduction in the number of transverse components and T‐tubular openings on SS, with areas devoid of T‐tubules within the cardiomyocytes (He *et al*. 2001);
2. a greater proportion of TATS elements running in the longitudinal and oblique directions (Louch *et al*. 2006; Swift *et al*. 2008; Wagner *et al*. 2012);
3. increased mean T‐tubular diameter and length (Ibrahim *et al*. 2012; Wagner *et al*. 2012).

Investigations of diseased T‐tubules have also been performed in intact hearts using *in situ* imaging that avoids any potential artefact related to cardiomyocytes isolation (Chen *et al*. 2015). Interestingly, in a rat model of left ventricular hypertrophy, *in situ* confocal imaging has initially revealed a localized T‐tubular remodelling, that then spreads from the left to the right ventricles together with the progression of the disease towards overt heart failure (Wei *et al*. 2010).

In HF ventricular cardiomyocytes, the above‐mentioned geometrical alterations have been linked to Ca^2+^ release abnormalities and dyssynchrony (Litwin *et al*. 2000; Louch *et al*. 2004, 2006). The disorganization of the TATS produces an array of repositioned DHPRs on the SS and leaves a large number of ‘orphaned’ RyRs at the Z‐lines (RyR clusters that become physically separated from their DHPRs partners) (Gomez *et al*. 2001; Song *et al*. 2006). The orphaned RyR release Ca^2+^ with variable latencies, as local Ca^2+^ elevation occurs after diffusion of Ca^2+^ from nearby normally triggered release units, i.e. *propagated‐CICR*. Thus, orphaned RyR2 channels are a major culprit for local Ca^2+^ release delay and reduced Ca^2+^ release synchrony in failing cells (Song *et al*. 2006), as well as in the case of experimental detubulation obtained through acute osmotic shock (Brette *et al*. 2006). Local Ca^2+^ release desynchronization promotes a slowing and broadening of the overall Ca^2+^ transient and thus can directly contribute to depressed contractility and prolonged contraction kinetics in heart failure (Sipido *et al*. 1998; Lyon *et al*. 2009). As a proof of concept, we recently demonstrated that a loss of TATS after experimental acute detubulation leads to a depressed contractile force and slower twitch kinetics (Ferrantini *et al*. 2014a), that can be reversed by pharmacologically enhancing the propagation of CICR to orphan RyR2 clusters with RyR2 Ca^2+^‐sensitizers (Oyehaug *et al*. 2013; Crocini *et al*. 2014b). Hence, the presence and the level of activity of orphaned RyR2 appear to be a major determinant of myocardial contractile performance. In addition, even though the integrity of dyads is maintained, failing cardiomyocytes exhibit a complex array of functional abnormalities affecting both RyRs and DHPRs. For instance, disrupted modulation of RyR gating in HF is also caused by excessive phosphorylation (Curran *et al*. 2010), decreased binding to the regulatory protein FKBP12.6 (Ono *et al*. 2000), and redox modification (Terentyev *et al*. 2008). Moreover, redistribution of DHPRs away from the T‐tubules has been recently reported in HF models (Bryant *et al*. 2015; Sanchez‐Alonso *et al*. 2016), confirming altered ECC at TATS level in HF. In the next paragraph, we will highlight how newly found electrical alterations of the TATS may contribute for local Ca^2+^ release desynchronization in remodelled ventricular cardiomyocytes.

## Electrical defects of the TATS probed by random‐access multiphoton microscopy

A number of studies have speculated that the AP at the T‐tubules is longer than at the SS in ventricular cardiomyocytes based on differences in channel density and electrochemical cation gradients between the two membrane domains (Tidball *et al*. 1988; Clark *et al*. 2001; Swift *et al*. 2006). Inversely, the tight electrical coupling between the two membrane compartments would guarantee a uniform AP duration throughout the sarcolemma in individual cardiomyocytes. The uniformity of the AP across the whole sarcolemma has been indeed mathematically (Pasek *et al*. 2003) and experimentally (Bu *et al*. 2009) proven, but the consequences of structurally disorganized TATS on AP propagation have been unclear for a long time. We have developed a random‐access multiphoton (RAMP) microscope that, in combination with fluorinated voltage‐sensitive dyes (VSD) (Yan *et al*. 2012), allows us to simultaneously assess the AP at different membrane domains within a cardiomyocyte. In detail, the RAMP microscope is provided with an ultrafast scanning head consisting of two acousto‐optic deflectors (AODs). AODs rapidly scan lines on different membrane segments with a commutation time of 4 μs between a line and the next. In a typical measurement, we probed 5–10 different membrane sites and the length of the scanned lines ranged from 2 to 10 μm with an integration time per membrane pass of 200 μs, leading to a temporal resolution of 0.4–2 ms. Using RAMP microscopy, we have confirmed that the tight electrical coupling between the T‐tubular system and the surface sarcolemma is ensured in intact isolated ventricular cardiomyocytes (Sacconi *et al*. 2012). In addition, we have demonstrated that in a rat model of post‐ischaemic HF, structurally remodelled TATS exhibit abnormal electrical activity:

1. failure of AP propagation: approximately 7% of T‐tubules (AP‐failing T‐tubules) did not show any voltage variation while a stimulated AP normally occurs on the surface sarcolemma and neighbour T‐tubules;
2. the presence of local spontaneous depolarizations that occur only in AP‐failing T‐tubules and do not propagate to the whole sarcolemma.

**Figure 1:**
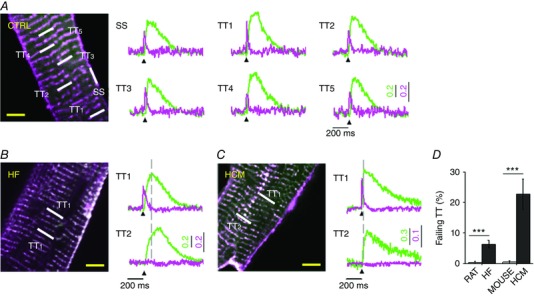
Action potential propagation and Ca^2+^ release in healthy and diseased cardiomyocytes *A*, two‐photon fluorescence (TPF) image of a control ventricular myocyte isolated from rat: sarcolemma in magenta stained with a voltage‐sensitive dye (di‐4‐ANE(F)PTEA) and cytoplasm in green with a fluorescent Ca^2+^ indicator ([Ca^2+^]_i_, GFP‐certified Fluoforte). On the right, normalized fluorescence traces (Δ*F*/*F*_0_) simultaneously recorded from the scanned sites indicated in white in image: surface sarcolemma (SS) and five T‐tubules (TTi). Membrane voltage (magenta) and [Ca^2+^]_i_ (green). *B*, TPF image of a stained rat ventricular myocyte isolated from a failing heart: membrane in magenta and [Ca^2+^]_i_ in green. On the right, average of 10 subsequent fluorescence traces (Δ*F*/*F*_0_) from the scanned lines indicated in the TPF image. Membrane voltage in magenta and [Ca^2+^]_i_ in green. The grey dashed line indicates the Ca^2+^ release time‐to‐peak measured in TT1. *C*, TPF image of a stained mouse ventricular myocyte isolated from a hypertrophic cardiomyopathy mouse: membrane in magenta and [Ca^2+^]_i_ in green. On the right, average of 10 subsequent fluorescence traces (Δ*F*/*F*_0_) from the scanned lines indicated in the TPF image. Membrane voltage in magenta and [Ca^2+^]_i_ in green. The grey dashed line indicates the Ca^2+^ release time‐to‐peak measured in TT1. AP is elicited at 200 ms (black arrowheads). Scale bar of 5 μm in orange on the TPF images. *D*, columns showing the percentage of electrically failing T‐tubules in CTRL rats, failing rats, CTRL mouse and HCM mouse. Data reported as means ± SEM. Data from 27 CTRL rat cells (124 TTs, *N* = 5); 59 rat HF cells (364 TTs, and 23 failing TTs, *N* = 9); 28 CTRL mouse cells (101 TTs, *N* = 10) and 66 HCM mouse cells (66 TTs, and 15 failing TTs, *N* = 7). Asterisks indicate significant differences (Student's *t* test, ^***^ *P* < 0.001). Figures and data reproduced with permission from Crocini *et al*. (2014b, 2016c).

The effects of these TATS electrical alterations on local Ca^2+^ release have been disclosed in a more recent work (Crocini *et al*. 2014b), in which rat ventricular cardiomyocytes were also stained with a fluorescent Ca^2+^ indicator. The RAMP microscope was used to simultaneously excite both dyes and, thanks to the large Stokes shift of the fluorinated VSD, the two components of the fluorescence signal were easily distinguished using appropriate optical tools. In this improved configuration, the RAMP microscope is capable of dissecting the spatiotemporal relationship between TATS electrical activity and Ca^2+^ release. In control cardiomyocytes, we found that the uniform AP propagation guarantees synchronous Ca^2+^ transients across the whole cell (Fig. 1 *A*). In HF, Ca^2+^ transients are globally delayed compared to control, but the electrical abnormalities further impair the local Ca^2+^ release. In fact, Ca^2+^ transients in correspondence with electrically failing T‐tubules show a significant additional delay likely to be due to a propagative Ca^2+^‐cascade from the neighbouring functioning sites (Fig. 1 *B*). Moreover, we observed that local spontaneous depolarizations occasionally trigger local Ca^2+^ release (voltage‐associated Ca^2+^ sparks, V‐sparks). V‐sparks may represent a novel pro‐arrhythmogenic phenomenon in HF setting. Finally, electrical defects can locally blunt β‐adrenergic signalling in HF (Crocini *et al*. 2016a).

Electrical properties of the TATS have been also evaluated in a mouse model of hypertrophic cardiomyopathy (HCM), expressing a human mutated cardiac troponin T (deletion of a codon at position 160 of the protein, cTnT Δ160E) that is associated with high risk of sudden cardiac death in patients (Pasquale *et al*. 2012; Coppini *et al*. 2014). Interestingly, in cTnT Δ160E ventricular cardiomyocytes, we observed about 20% of AP‐failing T‐tubules and consequent local Ca^2+^ release abnormalities (Fig. 1C and *D*), while TATS morphological alterations were minimal (Crocini *et al*. 2016c). This result suggests that the number of failing T‐tubules is not correlated with the degree of lost T‐tubular elements. Further support comes from our findings regarding acutely detubulated cardiomyocytes, in which a dramatic detachment of T‐tubules is associated with only 12% of failing T‐tubules among the remaining connected elements (Sacconi *et al*. 2012).

## Possible causes of electrical defects of the TATS

**Figure 2:**
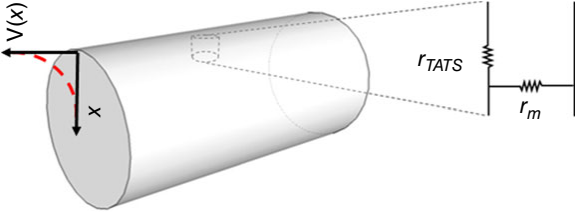
Cylinder representing an isolated rat cardiomyocyte Every infinitesimal volume of the TATS can be associated with an equivalent electrical circuit based on *r*_TATS_, and *r*_m_.

At first glance, the lack of detectable APs could suggest the absence of the channels responsible for membrane depolarization at the failing T‐tubules. Though a subcellular assessment of the protein expression has not been performed, membrane channel composition and density are unlikely to be affected by the osmotic shock in detubulated cells, where AP‐failing T‐tubules have been found. Further, the observation of spontaneous depolarizations in AP‐failing T‐tubules of HF cardiomyocytes encourages the assumption that voltage‐gated channels are still present. Thus, the reasons for electrical abnormalities must be sought elsewhere. When depolarization reaches a critical level (threshold), the cardiomyocyte responds in an active manner by opening voltage‐gated ion channels, producing an all‐or‐none response in the form of an AP. Yet, membrane potential variations elicit also passive (electrotonic) responses of the cellular membrane that do not need the opening of gated ion channels. Considering the AP propagation through the TATS, the extent of passive responses is only limited by the voltage drop due to the current flowing down the TATS (*r*_TATS_) and the dispersion of the current across the membrane (*r*_m_) (Fig. 2). The change in membrane potential δ*V*_m_ decays exponentially with distance and is expressed by: $V\;\left(x\right)=V_{0}\;e^{-\frac{x}{\lambda }}$ where λ is the membrane *space* or *length constant*, *x* is the distance from the site where AP is originated (the surface sarcolemma), and *V*_0_ is action potential amplitude at x=0. The length constant λ can be calculated as λ=rm/r TATS  and a theoretical estimation indicates it is ≅240μm (Pasek *et al*. 2008b). The maximum length of T‐tubules is about 25μm (Soeller & Cannell, 1999), much smaller than the length constant λ, meaning that the passive voltage drop of AP is of a few millivolts from the SS to the cell core. Thus, even if a failing T‐tubule were devoid of voltage‐gated channels, it should still exhibit passive responses and electronically propagate the AP.

Based on the considerations above, the underlying causes for electrical failure at the level of single T‐tubules seem to relate to ultrastructural alterations such as local strictures of the T‐tubular lumen. Strictures may produce a local drop of conductivity at the tubular level and thus a sudden interruption of electrical propagation; this alteration could also represent the earlier event of T‐tubular detachment.

Here, we propose two very different approaches to address this issue: (i) a correlative study, and (ii) an assessment of the diffusion of the TATS.

## Future investigative strategies

Electron microscopy (EM) is probably the first‐choice tool for the fine exploration of biological structures. In fact, the spatial resolution of EM is superior to optical microscopy and can extend to 1 nm, assuring a proper reconstruction of the detailed T‐tubular ultrastructure. However, EM provides a very limited field of view and, most importantly, requires fixed samples, which undermines the possibility of studying the electrical function of T‐tubules. Consequently, a combined strategy could be employed: first, the failing T‐tubule can be located by using the RAMP microscope and then, the ultrastructure of that particular failing T‐tubule could be accurately outlined exploiting the nanometric resolution of the EM. The integration of multiple systems for a multi‐level study of the same biological event is called *correlative microscopy*. Correlative microscopy has been widely used for a variety of investigations (Silvestri *et al*. 2014; Allegra Mascaro *et al*. 2015) and EM technological advances in EM, for instance the serial block face (SBF) scanning EM (Pinali & Kitmitto, 2014), allow for a three‐dimensional reconstruction of the sample without manual sectioning. We envision two main challenges that need to be overcome for the study of failing T‐tubules by correlative microscopy: (i) the sample processing procedure, and (ii) the three‐dimensional localization of the failing T‐tubule. Once functional data are obtained in living cardiomyocytes, the sample has to be processed to become available for structural investigations by EM. Cells are fixed and treated with different solutions before getting embedded in resin. Such a long pipeline produces unavoidable deformation of the sample that needs to be considered. Another issue is the three‐dimensional localization of the exact same T‐tubule with the different imaging methods. Fiducial marks are usually branded around the region of interest and, recently, marks have been obtained using a pulsed near‐infrared laser (Bishop *et al*. 2011). These marks are fluorescent and can also be photo‐oxidized to generate electron contrast, guiding re‐identification of previously imaged T‐tubules.

A totally different approach for studying T‐tubular ultrastructure could exploit the diffusion within the TATS. Diffusion refers to the process by which mass is transferred from a region of high concentration to a region of low concentration. Molecules that are impermeable to the membrane can diffuse in the extracellular space including the TATS lumen. Of course, the TATS represents a restrictive diffusion space and the smaller the accessible section the slower is the diffusion process. A diffusion coefficient *D*'can be defined for T‐tubules as D′=Dσπr TT 2, where σ is the superficial density of T‐tubules and *r*_TT_ is the T‐tubular radius, i.e. the ratio between the T‐tubular cross‐sectional area and the total area of the cellular surface. We hypothesize that diseased cardiomyocytes characterized by failing T‐tubules display local structural alterations that suddenly affect the passive voltage drop of AP as well as the availability of space for molecules to diffuse into the TATS lumen. Thus, studying the diffusion of fluorescent molecules from extracellular space to the TATS could provide information regarding the geometry of the system itself.

In conclusion, we propose, here, two conceptually different strategies to investigate the biophysical reasons underlying T‐tubular electrical defects. The development of non‐invasive ultrafast technologies for imaging and optically controlling cardiac function (Crocini *et al*. 2014a, b) has indeed provided novel insights into cardiac pathophysiology and has consequently generated new challenges. The discovery of electrically failing T‐tubules in pathologies is an extremely intriguing phenomenon that dramatically affects ECC machinery and dulls signalling responses. We believe that unveiling the foundations of electrical abnormalities not only could help development of therapeutic tools to improve cellular electro‐mechanics, but also represents an exceptionally fascinating challenge for biophysicists.

## Additional information

### Competing interests

The authors declare no conflict of interest.

### Author contributions

All authors have approved the final version of the manuscript and agree to be accountable for all aspects of the work. All persons designated as authors qualify for authorship, and all those who qualify for authorship are listed.

### Funding

This work has received funding from the European Union's Horizon 2020 research and innovation programme under grant agreement no 654148 Laserlab‐Europe. This research project has been also supported by National Institutes of Health (NIH Grant: R01 EB001963), by the Italian Ministry for Education, University and Research in the framework of the Flagship Project NANOMAX, by the Italian Ministry of Health (WFR GR‐2011‐02350583), by Telethon–Italy (GGP13162), by Ente Cassa di Risparmio di Firenze (private foundation), and by Regione Toscana (PAR‐FAS Salute 2014, ‘TORSADE’ project).
